# A Novel Antibiotic Spacer for Significant Proximal Femoral Loss - Surgical Technique

**DOI:** 10.2174/1874325001711010508

**Published:** 2017-05-31

**Authors:** David Shields, Roderick Kong, Sanjay Gupta, Ashish Mahendra

**Affiliations:** Department of Musculoskeletal Oncology, Glasgow Royal Infirmary, 84 Castle Street, Glasgow, G4 0ET Scotland, United Kingdom

**Keywords:** Infection, Bone loss, Revision, Hip, Femur, Antibiotics

## Abstract

**Background:**

Infections of proximal femora with prosthetic implants in situ have long been a major concern in orthopedic surgery. The gold standard in the management of infected proximal femurs in the presence of prosthetic implants has traditionally been a two-stage revision. However, this is challenging in the setting of extensive bone loss.

**Methods:**

A 3 case series of such infections leading to extensive loss of the proximal femur is presented. We specifically describe our technique of debriding the infected segments as well as utilization of a trochanteric slide osteotomy to resect the femur.We also demonstrate preparation of the “pseudoacetabulum” and femoral component with an antibiotic spacer.

**Conclusion:**

The high cost of such a procedure is offset by reduction in time spent in hospital. The spacer also helps to allow mobilization by partial weight bearing on a stable femoral component and provide pain control which improves quality of life as compared to prolonged intravenous antimicrobial therapy.

## INTODUCTION

Oncology patients are at high risk of infection due to a combination of factors including destruction from the neoplastic process, extensive soft tissue resection, cavitation/dead-space and neo-adjunctive therapies. The primary reconstruction of bones and joints using a combination of endoprosthetic replacements and intercalary prostheses are complex. Thus, when infection ensues, effective strategies are needed for managing such cases to minimise the loss of prosthesis or amputation [1].

Infection of the proximal femur in the presence of prosthetic implants can lead to significant functional detriment and frequently necessitates two-stage revision of the components [2-6]. If there is significant involvement of the femoral diaphysis, or a femoral stem is well fixed, radical resection of the proximal femur may be necessary. In order to optimize wound healing and prevent shortening, a stable antibiotic eluting femoral spacer can be introduced. When bone loss is minimal and muscular attachments are maintained, a simpler Girdlestone procedure with a cement ball or shaped prosthesis can be used. However, when larger debridements are necessary, achieving length and stability can be challenging. In our department we have developed a novel technique of attaining these goals by forming a temporary proximal femoral replacement from an antibiotic ‘pseudoacetabulum’ articulating with an intramedullary nail surrounded by antibiotic cement.

In this article we present the technique and 3 case examples where we have successfully applied this method.

## TECHNIQUE

During preoperative planning the amount of femur requiring resection is estimated from measurements on radiographs. We liaise with our microbiology colleagues to tailor the appropriate antibiotic regimen (both intravenous and cement). Theatre equipment is prepared including a large head monoblock hemiarthroplasty (such as an Austin-Moore), femoral head sizers and deployment of fluoroscopy.

**Fig. (1):**
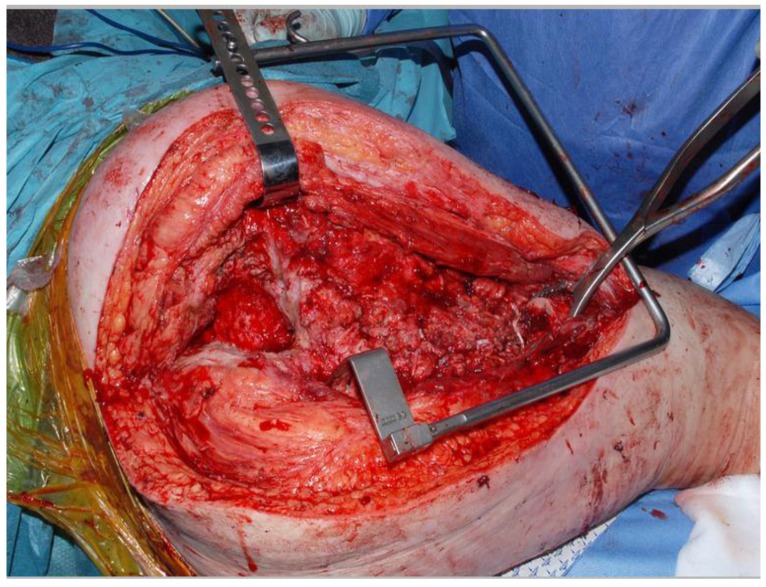
Post resection of prosthesis and debris. Note reamed acetabulum on the left.

Antibiotics are introduced routinely at induction of anesthesia. The previous wound is opened and dead, dying or infected tissue is debrided (Fig. **1**). A trochanteric slide osteotomy (similar to the technique described by Ganz [7]) is favored to maintain continuity of the abductors with the vastus lateralis. The femur is resected at the necessary level and any remaining muscle attachments are released. The femur is marked for rotational alignment prior to the femoral osteotomy. The acetabulum is cleared of any debris and an acetabular reamer is used with caution to induce clean and bleeding surfaces.

### 1 Pseudoacetabulum Preparation

**Fig. (2):**
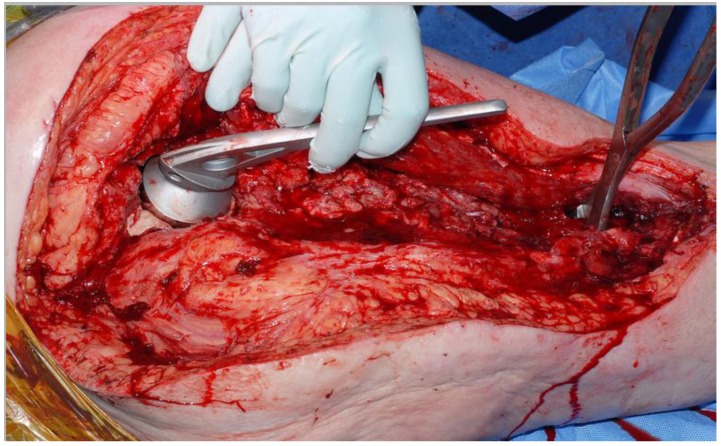
Creation of pseudoacetabulum by compressing a 56mm Austin-Moore into the cement.

A 60ml mix of cement (Refobacin^®^, Biomet) is mixed in a vacuum free environment (to maximize the porosity, and optimize elusion [8, 9]) and then rolled up and placed into the acetabular cavity. The head of a trial hemiarthroplasty is used to create an articulation for the femoral component whilst it sets, in neutral version (Fig. **2**). We find that lubricating and twisting the hemiarthroplasty with saline prevents the cement sticking to the prosthesis.

### 2 Femoral Preparation

Using the preoperative planning and measurements of the excised femoral section calculation of the length to restore is performed. The length from the centre of rotation of the femoral head to the distal femoral osteotomy site is noted and 7.5mm added to give the length of intramedullary nail used. This nail and a 100mm cephalomedullary screw are coupled and locked in position using the set screw available from our manufacturer. A line 7.5cm proximal to the tip of the nail is marked to indicate the limit of the cement. The remaining distal femoral canal is widened with sequential flexible reamers to just beyond the length of nail to be buried distally. Should there be a substantially longer distal femoral section remaining, a length greater than 7.5cm of nail may be inserted into the femur.

**Fig. (3):**
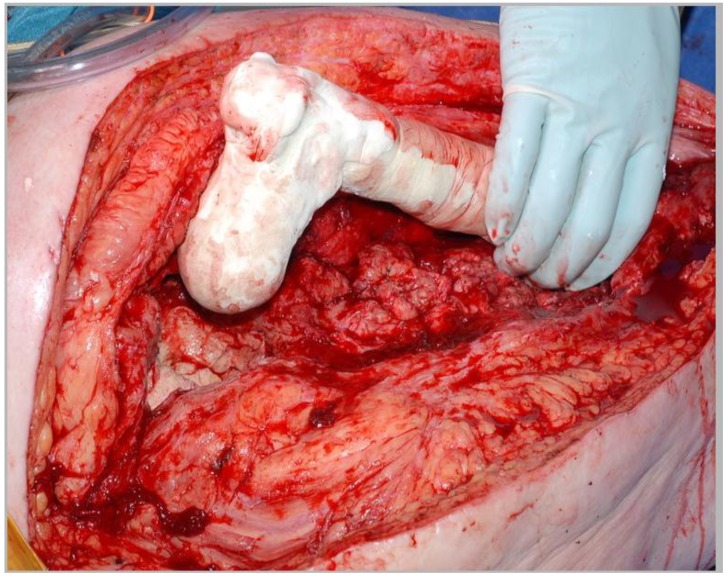
Cement formed around the nail is inserted into the femur.

The rough outline of the femur is formed using 200mls of cement, with a femoral head formed by hand and sized (using the sizing guides) to several millimeters in diameter smaller than the chosen hemiarthroplasty (Fig. **3**). Rotation of the component is marked and cement is allowed to set prior to trial reduction so as to avoid inducing secondary polymerization between the new femoral head and the pseudoacetabulum.

Once the cement has set and the femoral length trialed (long enough to avoid shortening of tissues in the interval before second stage revision, but not so long as to put tension on the wound or prevent reduction) distal locking is performed in a routine manner with intraoperative fluoroscopic guidance.

Following surgery, patients are permitted to partially weight bear and to commence 6 weeks of tailored intravenous antibiotic therapy as an outpatient.

## Case 1

Patient 1 is a 59 year old man who had a hybrid THR for osteoarthritis. After 6 years, he developed a spontaneous infection and underwent a 2 stage revision for proven infection (Group B Streptococcus).

**Fig. (4):**
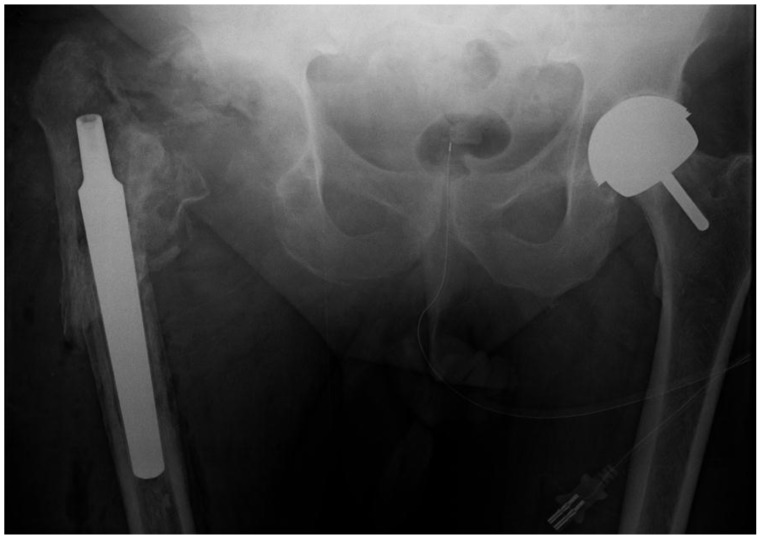
Pelvic x-ray showing the retained femoral stem prior to the second revision.

The patient was diagnosed with a recurrent deep infection based on elevated inflammatory markers and abscess formation. Intraoperatively, a periprosthetic fracture occurred while attempting to remove the well-fixed stem (Fig. **4**).

**Fig. (5):**
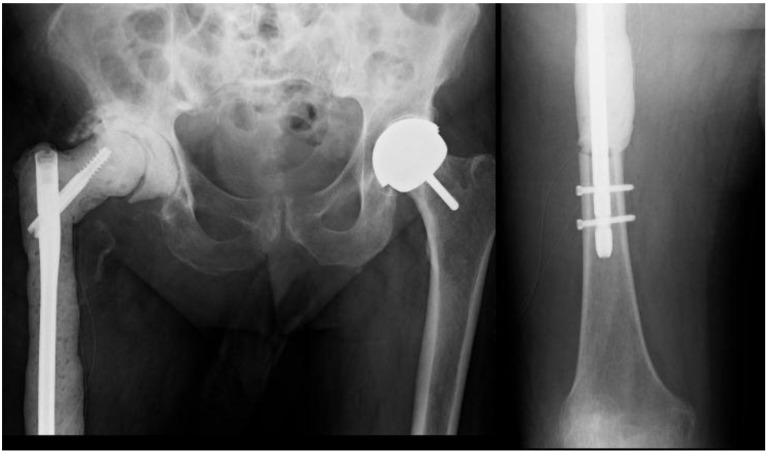
Postoperative X-rays showing the temporary antibiotic spacer construct. Note the antibiotic cement does not surround the nail within the bone, as opposed to a pre-constructed antibiotic nail.

A decision was reached to return at a later point to sacrifice the proximal femur during the first of another 2 stage revision to a proximal femoral replacement on this occasion. The patient had debridement and excision of the proximal femur as per this described technique (Fig. **5**).

**Fig. (6):**
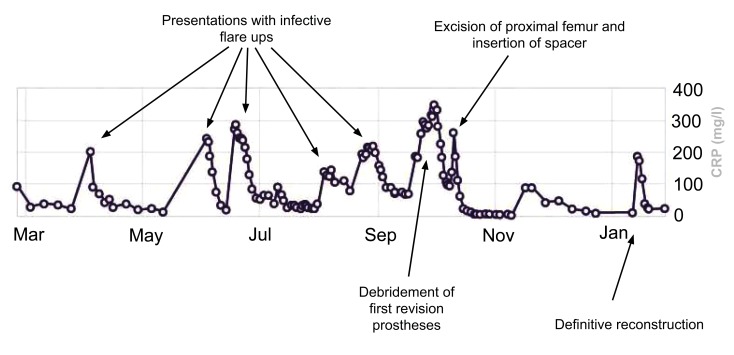
CRP trend in the pre and post operative period.

**Fig. (7):**
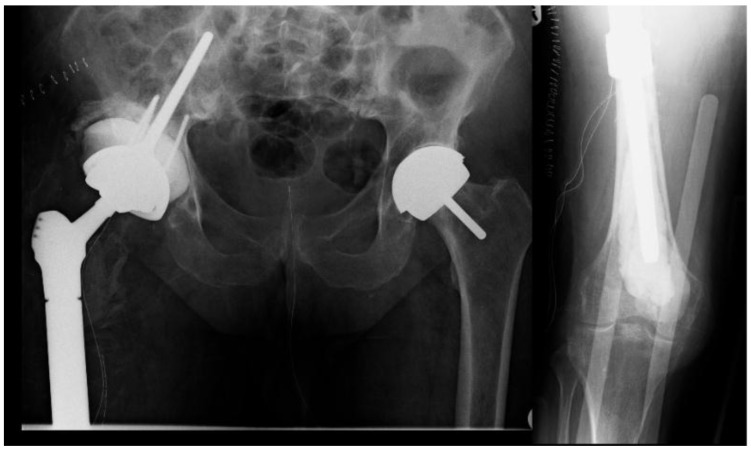
Definitive reconstruction with proximal femoral replacement and cone pelvis reconstruction.

The inflammatory markers subsequently normalized (Fig. **6**). After a 3 month period, 6 weeks of which outpatient intravenous antibiotics were given, the definitive reconstruction with a cone hemipelvis and proximal femoral replacement was performed (Fig. **7**).

## Case 2

**Fig. (8):**
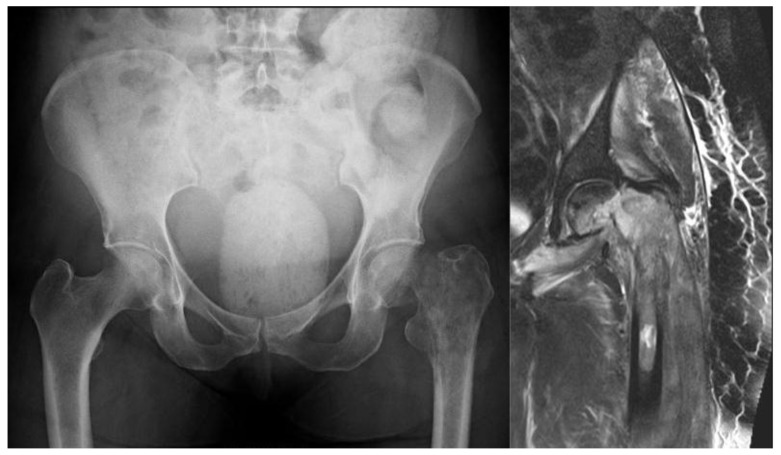
X-ray and MRI showing pathological fracture with involvement of proximal femoral diaphysis.

**Fig. (9):**
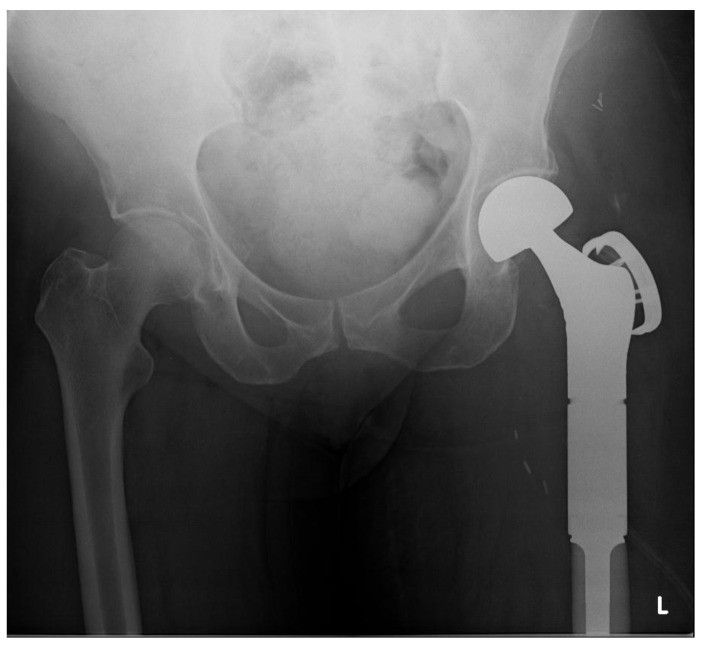
Proximal femoral replacement as management of the pathological fracture.

A 71-year old female presented with a pathologic intracapsular fracture of the left proximal femur (Fig. **8**) which was diagnosed as a primary diffuse large B-cell lymphoma amenable to curative therapy. A proximal femoral resection and replacement with a bipolar head was performed (Fig. **9**).

**Fig. (10):**
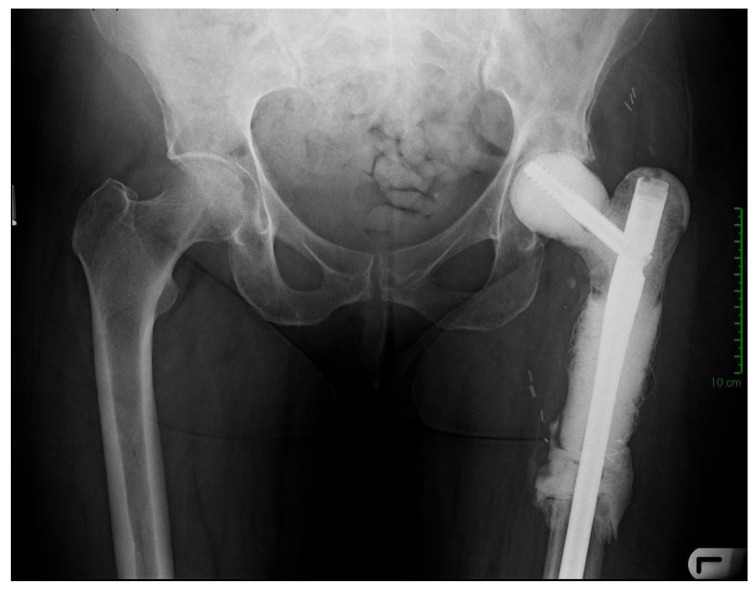
Postoperative x-ray following 1st stage revision.

**Fig. (11):**
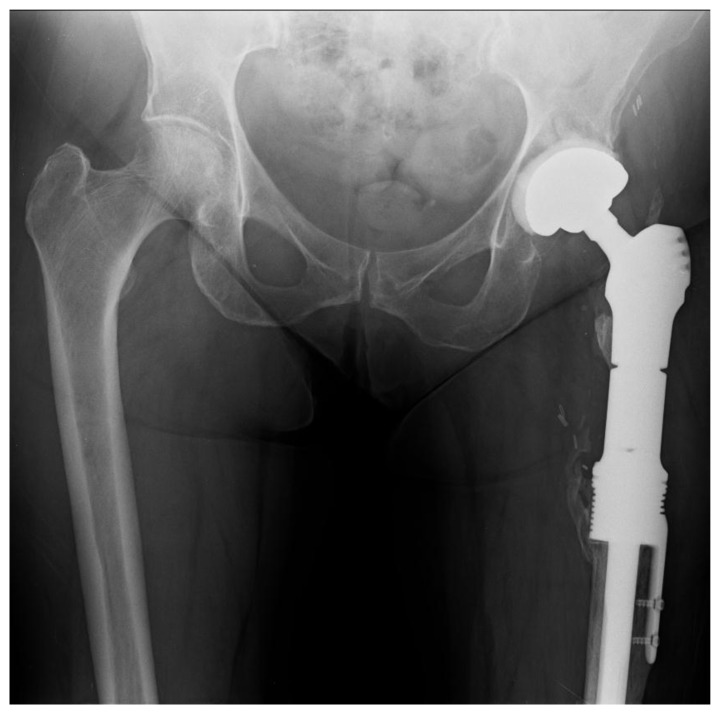
X-ray 3 years following 2nd stage revision with no evidence of loosening and signs of bone integration onto the prosthesis.

Eighteen months later, the patient developed progressive pain in the left thigh and was found to have elevated inflammatory markers. A subcutaneous abscess with Enterococcus and coagulase negative Staphylococcus was diagnosed. Despite an irrigation and debridement, her symptoms persisted and a two-stage revision was performed using the described technique (Fig. **10**). A silver-coated proximal femoral replacement was implanted 3 months later after normalization of inflammatory markers and withdrawal of antibiotics (Fig. **11**).

## Case 3

**Fig. (12):**
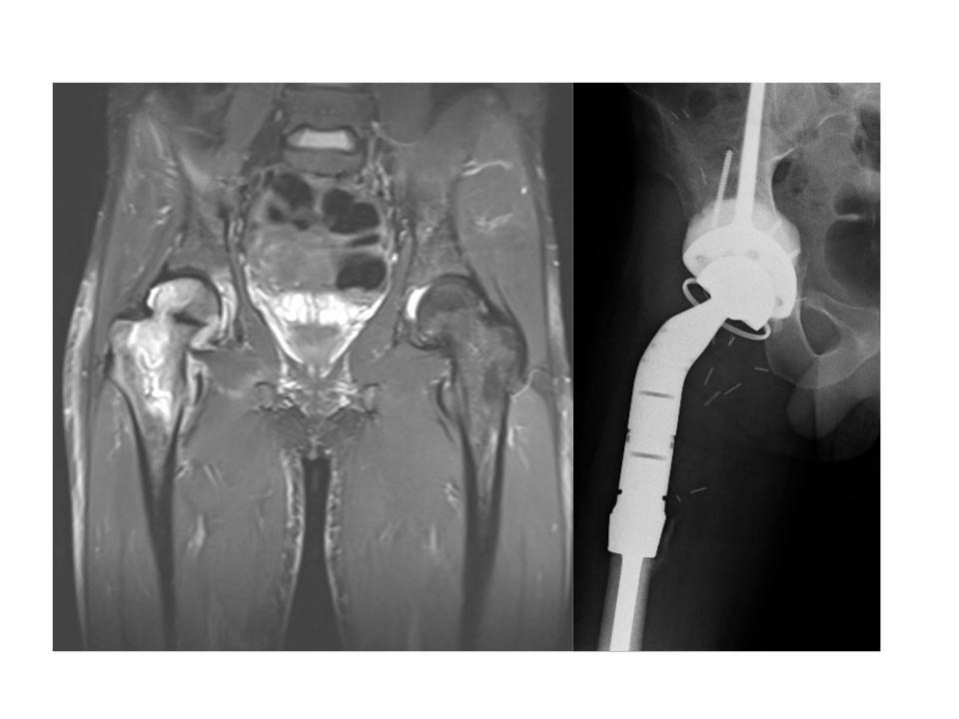
Preoperative MRI and postoperative x-ray of the pleomorphic sarcoma reconstructed with a coned pelvis.

A 25 year old man developed a high grade pleomorphic sarcoma of the right proximal femur extending into the hip joint. This was curatively excised with extra-articular resection and reconstructed with a coned pelvis with a subsequent unremarkable recovery (Fig. **12**).

**Fig. (13):**
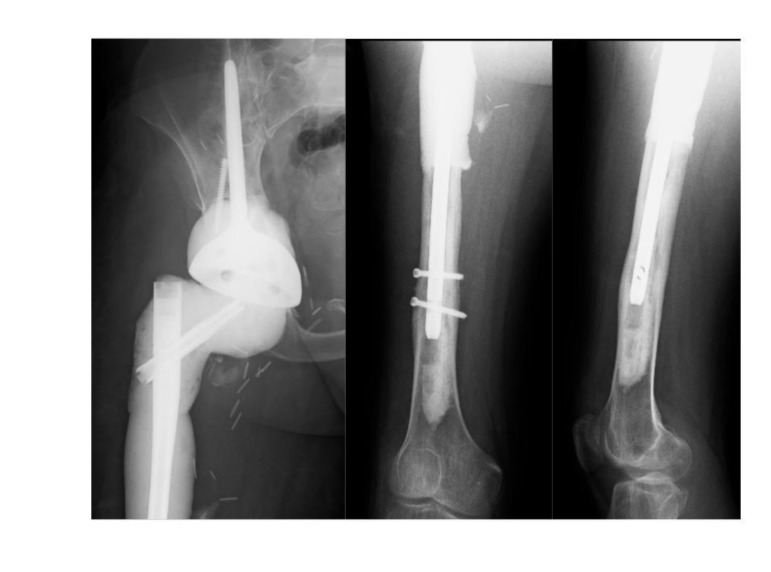
Post operative x-ray of femoral spacer. Note long segment of nail buried into void from previous femoral component stem and preservation of coned pelvis.

Almost 3 years following treatment his wound became inflamed and a deep abscess developed, which on aspiration grew staphylococcus epidermidis. The patient had the first stage of his revision completed (Fig. **13**). As the infection was found to be contained relatively distally, the coned pelvic segment was preserved.

## DISCUSSION

The current standard of care for management of established infection in the presence of prosthesis is a 2 stage revision with use of an antibiotic spacer between stages [2-6]. This technique was originally described by Insall in 1983, who noted the necessity of debridement and use of interim antibiotics [10].

Dealing with bone loss in the presence of infection is challenging in any circumstance, however in the lower limb inferring stability to maintain a level of mobility and optimizing pain control and nursing care becomes even more difficult.

Two-stage revision with an antibiotic spacer has been shown to reduce rates of reinfection [11]. It creates a more stable environment for healing and prevents soft tissue contractures. However, arthrofibrosis can occur between explantation of the original implant and reimplantation of the definitive prosthesis. We believe that the use of this construct infers stability, maintains soft tissue tension and as there is a longer lever arm with more mobility, arthrofibrosis is minimized. Furthermore the induction of a large biomembrane, similar to the masquelet technique, is beneficial to the ongoing biological activity following the definitive revision [12, 13].

We have evaluated simpler and perhaps cheaper alternatives, but have found this one most effective. Although a traction pin or elongated cement spacer are options, this technique infers stability due to the intramedullary nail having been designed for load bearing and the distal locking screws preventing rotation.

Although expensive (approximately £800), the long intramedullary nail is crucial for stability in this technique. These operations are frequently 3rd/4th down the line of a very expensive healthcare episode [14-16]. The patients will have spent weeks/months in hospital and will be tormented with not only a reduction in function/pain/malaise than before their primary joint, but also are facing the psychological distress of the impending reality of long term disability. Anything that gives our patient group a solid component to at least partially bear weight brings quality of life, better pain control and potentially shorter inpatient stay which saves money (current Department of Health estimates in the region of £400 per bed per day).

Due to the topical nature of the antibiotic application, and the low volume measurable in the blood, high concentrations of antibiotics can be used. In these cases 1g of vancomycin per 10ml of cement has been used as has been suggested in the literature [17]. Care should be taken if using lower doses, as this can give rise to antibiotic resistance [18, 19]. In our cases, we used wound drains, to allow drainage of any excess hematoma. However, the balance between draining antibiotic rich seroma and hydrostatic pressure on the wound should be considered. Although the majority of antibiotics are eluted within the first few hours to several days, studies on this combination of cement and antibiotics have demonstrated that the level of antibiotics remain bioactive and detectable, albeit at a much smaller dose at beyond 80 days [20].

## CONCLUSION

A custom construct of antibiotics and intramedullary femoral nail as outlined in this article is a safe and effective means of local antibiotic application for 2 stage revision for infection and large segment femoral bone loss.

## References

[1] Morii T., Morioka H., Ueda T., Araki N., Hashimoto N., Kawai A., Mochizuki K., Ichimura S. (2013). Deep infection in tumor endoprosthesis around the knee: a multi-institutional study by the Japanese musculoskeletal oncology group.. BMC Musculoskelet. Disord..

[2] Hofmann A.A., Goldberg T., Tanner A.M., Kurtin S.M. (2005). Treatment of infected total knee arthroplasty using an articulating spacer: 2- to 12-year experience.. Clin. Orthop. Relat. Res..

[3] Meek R.M., Masri B.A., Dunlop D., Garbuz D.S., Greidanus N.V., McGraw R., Duncan C.P. (2003). Patient satisfaction and functional status after treatment of infection at the site of a total knee arthroplasty with use of the PROSTALAC articulating spacer.. J. Bone Joint Surg. Am..

[4] Hsieh P.H., Chen L.H., Chen C.H., Lee M.S., Yang W.E., Shih C.H. (2004). Two-stage revision hip arthroplasty for infection with a custom-made, antibiotic-loaded, cement prosthesis as an interim spacer.. J. Trauma.

[5] Pitto R.P., Spika I.A. (2004). Antibiotic-loaded bone cement spacers in two-stage management of infected total knee arthroplasty.. Int. Orthop..

[6] Hofmann A.A., Goldberg T.D., Tanner A.M., Cook T.M. (2005). Ten-year experience using an articulating antibiotic cement hip spacer for the treatment of chronically infected total hip.. J. Arthroplasty.

[7] Ganz R., Gill T.J., Gautier E., Ganz K., Krügel N., Berlemann U. (2001). Surgical dislocation of the adult hip a technique with full access to the femoral head and acetabulum without the risk of avascular necrosis.. J. Bone Joint Surg. Br..

[8] Frommelt L., Kuhn K.D. (2006). “Properties of bone cement: antibiotic loaded cement,” in The Well-Cemented Total Hip Arthroplasty, part II..

[9] Lewis G., Janna S., Bhattaram A. (2005). Influence of the method of blending an antibiotic powder with an acrylic bone cement powder on physical, mechanical, and thermal properties of the cured cement.. Biomaterials.

[10] Insall J.N., Thompson F.M., Brause B.D. (1983). Two-stage reimplantation for the salvage of infected total knee arthroplasty.. J. Bone Joint Surg. Am..

[11] Garvin K.L., Hanssen A.D. (1995). Infection after total hip arthroplasty. Past, present, and future.. J. Bone Joint Surg. Am..

[12] Masquelet A.C. (2003). Muscle reconstruction in reconstructive surgery: soft tissue repair and long bone reconstruction.. Langenbecks Arch. Surg..

[13] Masquelet A.C., Begue T. (2010). The concept of induced membrane for reconstruction of long bone defects.. Orthop. Clin. North Am..

[14] Klouche S., Sariali E., Mamoudy P. (2010). Total hip arthroplasty revision due to infection: a cost analysis approach.. Orthop. Traumatol. Surg. Res..

[15] Vanhegan I.S., Malik A.K., Jayakumar P., Ul Islam S., Haddad F.S. (2012). A financial analysis of revision hip arthroplasty: the economic burden in relation to the national tariff.. J. Bone Joint Surg. Br..

[16] Kallala R.F., Vanhegan I.S., Ibrahim M.S., Sarmah S., Haddad F.S. (2015). Financial analysis of revision knee surgery based on NHS tariffs and hospital costs: does it pay to provide a revision service?. Bone Joint J..

[17] Jiranek W.A., Hanssen A.D., Greenwald A.S. (2006). Antibiotic-loaded bone cement for infection prophylaxis in total joint replacement.. J. Bone Joint Surg. Am..

[18] Moojen D.J., Hentenaar B., Charles Vogely H., Verbout A.J., Castelein R.M., Dhert W.J. (2008). *In vitro* release of antibiotics from commercial PMMA beads and articulating hip spacers.. J. Arthroplasty.

[19] Hendriks J.G., van Horn J.R., van der Mei H.C., Busscher H.J. (2004). Backgrounds of antibiotic-loaded bone cement and prosthesis-related infection.. Biomaterials.

[20] Stevens C.M., Tetsworth K.D., Calhoun J.H., Mader J.T. (2005). An articulated antibiotic spacer used for infected total knee arthroplasty: a comparative in vitro elution study of Simplex and Palacos bone cements.. J. Orthop. Res..

